# From fox to fork? *Toxocara* contamination of spinach grown in the south of England, UK

**DOI:** 10.1186/s13071-023-05674-8

**Published:** 2023-02-02

**Authors:** Sara R. Healy, Eric R. Morgan, Joaquin M. Prada, Martha Betson

**Affiliations:** 1 School of Veterinary Medicine, University of Surrey, Daphne Jackson Road, Guildford, GU2 7AL Surrey UK; 2 Institute for Global Food Security, Queen’s University, Biological Sciences Building, 19 Chlorine Gardens, Belfast, BT9 5DL UK

**Keywords:** *Toxocara*, Vegetables, Farms, Food safety, Zoonosis

## Abstract

**Background:**

*Toxocara canis* and *Toxocara cati* are intestinal parasites of dogs, cats and foxes, with infected animals shedding eggs of the parasite in their faeces. If humans accidentally ingest embryonated *Toxocara* spp. eggs from the environment, severe clinical consequences, including blindness and brain damage, can occur. Previous work has demonstrated the presence of *Toxocara* spp. eggs on vegetable produce grown in the UK, but only in small-scale community gardens. The aim of this study was to determine whether *Toxocara* spp. eggs are also present on vegetables grown on commercial farms in the UK, which supply produce to a greater number of people.

**Methods:**

A total of 120 samples (300 g each) of spinach (*Spinacia oleracea*) were collected across four farms in the south of England, UK. The samples were processed using a sieving approach followed by multiplex quantitative polymerase chain reaction analysis.

**Results:**

Overall, 23.0% of samples were positive for *T. canis* (28/120; 95% confidence interval 16.7–31.7%) and 1.7% for *T. cati* (2/120; 95% confidence interval 0.5–5.9%). There was a statistically significant difference in the number of positive samples between farms (*P* = 0.0064). To our knowledge, this is the first report of the isolation of *Toxocara* spp. from vegetables grown on commercial farms in the UK.

**Conclusions:**

The results of this study highlight the requirement for the thorough washing of vegetables prior to their consumption, especially those such as spinach which may be eaten without first peeling or cooking, and effective farm biosecurity measures to minimise access to farmland by definitive host species of *Toxocara* spp.

**Graphical Abstract:**

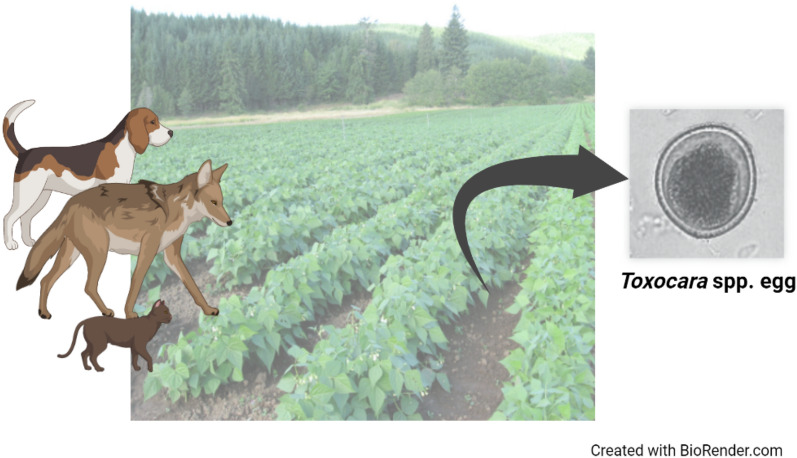

## Background

*Toxocara canis* and *Toxocara cati* are zoonotic roundworm parasites which reside in the intestinal tracts of infected dogs, cats and foxes. Large numbers of *Toxocara* spp. eggs can be shed in the faeces of infected animals [1], which subsequently contaminate the environment and become infective. If embryonated *Toxocara* spp. eggs are ingested by animal species other than their definitive hosts, or a human, the larvae cannot complete their development and instead migrate to a number of different locations in the body. In humans, this can result in severe pathological and debilitating clinical manifestations, including blindness and neurological disorders [2].

It has been known for some time that soil contaminated with infective *Toxocara* spp. eggs can lead to human infection following accidental ingestion, but more recent work has also shown the presence of eggs on produce destined for human consumption [3, 4]. For example, *Toxocara* spp. eggs were detected on 4.8% of sampled vegetables in a study undertaken in the Philippines, with leafy vegetables found to be most contaminated [5], and in a Brazilian study, 17.8% of sampled lettuces (*Lactuca sativa*) were found to be positive for *Toxocara* spp. eggs [6]. Additionally, two recent systematic reviews and meta-analyses evaluating the global incidence of the helminthic contamination of fruits and vegetables reported pooled *Toxocara* spp. prevalences of 7% [7] and 6% [8], respectively. The fact that there is, to our knowledge, currently only one study from the UK on the contamination of vegetables with *Toxocara* spp. eggs, which was undertaken on produce grown on a small scale on public land [9], highlights the need for further research to assess UK-grown produce for these parasites.

The aim of this study was to determine whether produce grown in fields by larger-scale, commercial producers in the south of England, UK, could also be contaminated with *Toxocara* spp. eggs, and thus pose a risk to a larger number of consumers if eaten unwashed. Spinach (*Spinacia oleracea*) was selected for testing based on its availability from vegetable farms, its potential for raw consumption by humans, and its leafy nature, a characteristic which has been linked to enhanced soil capture and a higher likelihood of *Toxocara* spp. egg contamination [5, 10].

## Methods

### Sample collection

Four large-scale commercial vegetable farms (ranging in size from 16 to 62 ha) located in the southern counties of Surrey, Berkshire, Greater London, and Buckinghamshire, England, UK, were selected for the study. The sites were selected on the basis of their proximity to the laboratory to facilitate the rapid transportation of samples and reduce the risk of spoilage. A variety of fruits and vegetables were cultivated on each farm by conventional methods. Spinach was selected for this study as it is widely available from vegetable farms, and leafy vegetables are reported to be more prone to contamination with soil and parasitic eggs within the folds of their leaves [10]. In addition, spinach may be consumed without prior peeling or cooking, and thus poses a greater risk of parasitic infection if ingested without prior washing. Whilst *Toxocara* spp. have been isolated from lettuce [5, 11], lettuce was not selected for sampling in this study as it is not commonly cultivated in fields on commercial farms in the UK because of its susceptibility to pest damage, in particular that due to slugs and snails, and is more commonly grown under cover or in hydroponic conditions.

**Fig. 1:**
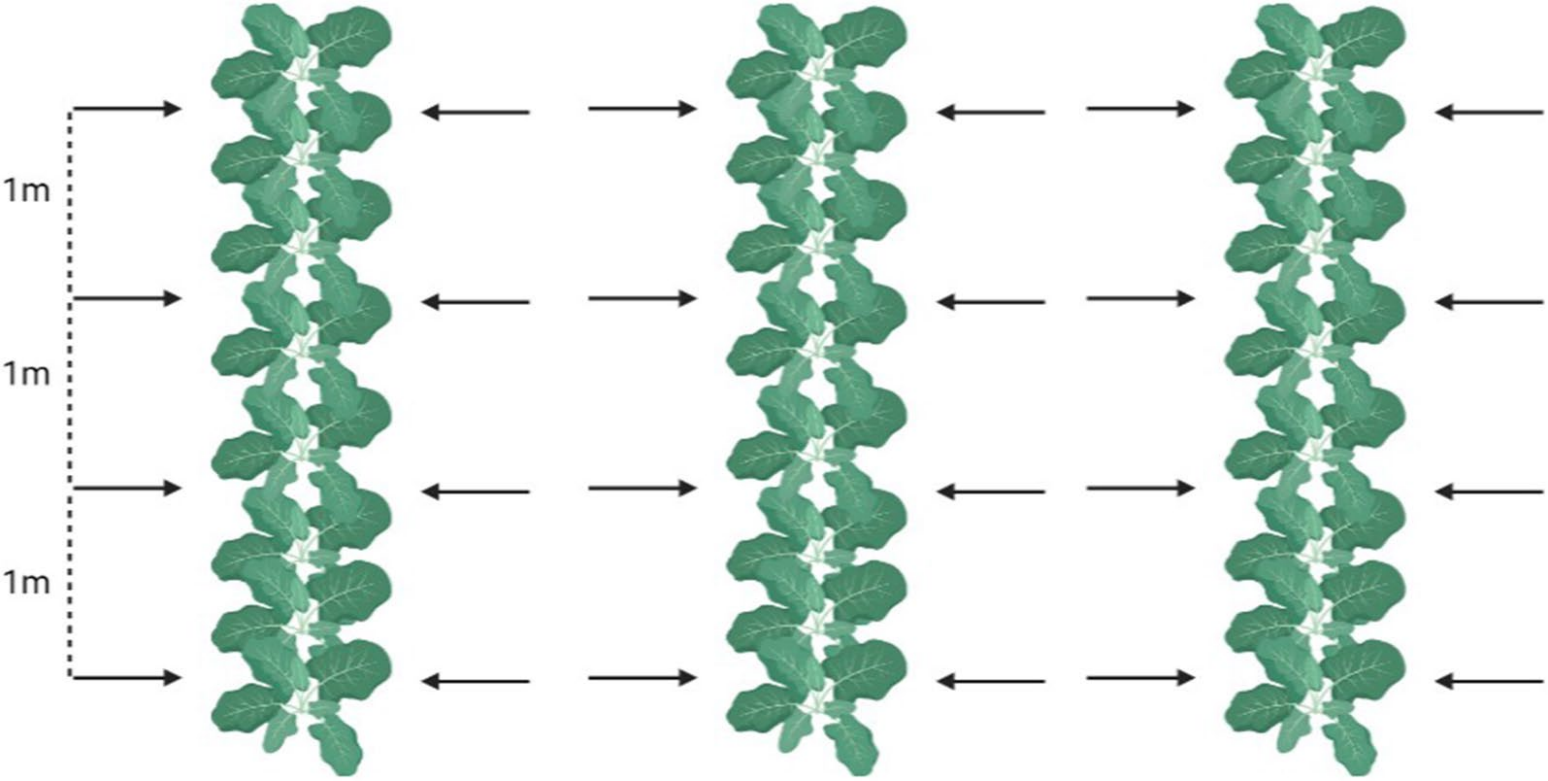
Diagram showing the sampling approach. Each row of spinach crop was sampled at approximately 1-m intervals (arrows). (Image created using BioRender.com)

Samples were collected during the crop growing season, between June and August, using a systematic sampling approach, with one large handful of leaves picked along each crop row at approximately 1-m intervals. Every available row of spinach crop in the fields was sampled (Fig. 1). Samples were transferred immediately to the laboratory for testing. A total of 120 samples of 300 g of leaves (approximately 36 kg in total) was tested, with a similar number of samples obtained per site depending on crop availability (33, 25, 35 and 27 samples, respectively). The sample size was calculated using WinEpi software (www.winepi.net) using a previously obtained prevalence value of 2.4% [9].

### Validation step: recovery of eggs from spiked vegetable samples

**Fig. 2:**
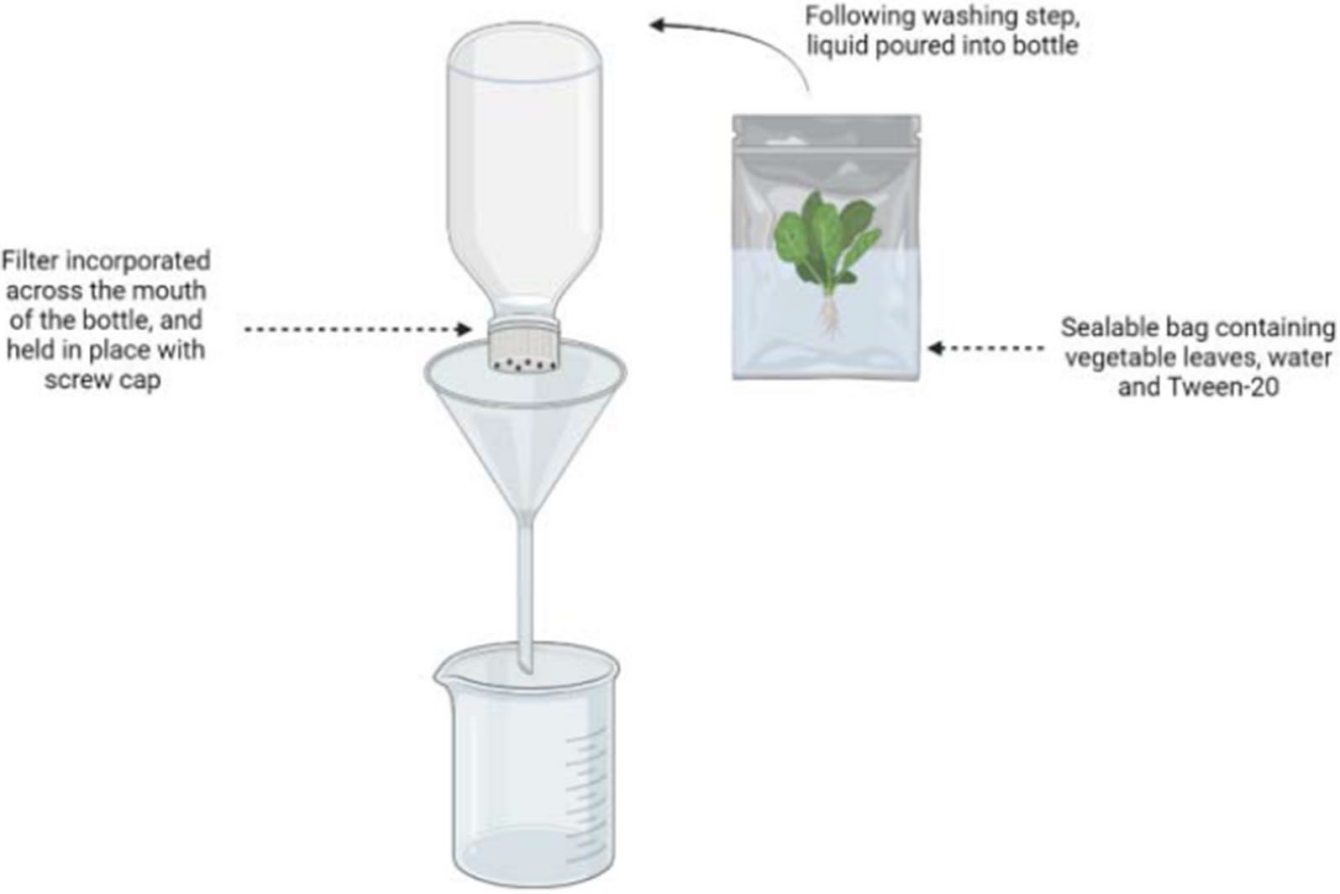
Diagram outlining the sieve system used to capture *Toxocara* spp. eggs from the vegetable washing solution. (Image created using BioRender.com)

In order to assess the efficiency of the methodology, egg-spiking experiments were undertaken using 300-g samples of lettuce (*L. sativa*) and spinach (*S. oleracea*). In brief, a simple sieving system was constructed, which was a modified version of the system used by Guggisberg et al. [11]. The system isolates parasitic eggs based on their size by concentrating them in a nylon filter with a pore diameter small enough to retain them. The pore size used here for *Toxocara* spp. eggs was 40 µm. For the ‘funnel’ component, a 1.5-L plastic bottle was cut into two, washed thoroughly, and the upper half of the bottle used with the neck/lid retained. The lid was removed and perforated and a single-use, nylon filter inserted into the top of the bottle before screwing the lid back on to secure it into place (Fig. 2).

*Toxocara* spp. eggs were harvested from adult worms retrieved from the small intestines of foxes during post-mortem examination, and stored in phosphate buffered saline at 4 °C until required. The eggs were enumerated before use and 25-µL aliquots containing a known concentration of eggs were applied to 300-g samples of lettuce and spinach in duplicate (4, 20 and 100 eggs). The samples were left to dry for 1 h before being placed into sealable plastic bags (30 × 40 cm). Negative control samples were also tested in duplicate.

The method of Guggisberg et al. [11] was used, with some modifications. A 500-mL volume of water containing 0.2% Tween 20 was added to each sealable plastic bag, the top sealed, and the bag shaken vigorously for 30 s. Each bag was left upright on the laboratory bench for 5 min to reduce the number of bubbles present, before snipping off one corner (approximately 5 mm) and pouring the liquid into the filtration funnel. The funnel was held inside a larger plastic funnel over the top of a 2-L beaker that was used to collect the filtrate. Single-use pipettes were used to free the filter of debris and avoid blockage. A 200-mL volume of water was poured into each sample bag for rinsing, and this liquid was also passed through the filtration system. A 100-mL volume of water was poured into the filtration system as a final rinse.

Once all the liquid had passed through, the filter was removed from the system and placed into a Falcon tube containing 45 mL of water. Each filter was rinsed with 5 mL of water, which was captured in the same Falcon tube. All plasticware, bags and filters were only used once, then discarded. The contents of each Falcon tube was centrifuged (1000 *g* for 5 min) and the supernatant discarded. The resulting pellet was transferred to a 1.5-mL Eppendorf tube. The Falcon tube was rinsed with a small volume of water, which was added to the Eppendorf tube. The samples were centrifuged (10,000×*g* for 3 min) and the supernatant removed. The final pellet was heat-treated at 90 °C for 10 min to weaken the outer egg structure prior to DNA extraction, as per Tyungu et al. [12].

### Molecular detection of* Toxocara* spp. eggs

DNA was extracted from the pellets using the Qiagen Powersoil Pro kit (Qiagen, USA) according to the manufacturer’s instructions, with 10 min of full-power bead beating in a TissueLyser LT (Qiagen, USA). The extracted DNA was subjected to quantitative polymerase chain reaction (qPCR) analysis as described by Tyungu et al. [12], using a 96-well C1000 Touch PCR thermal cycler (Bio-Rad, USA) with previously published probes for *T. canis* (5′-FAM-CCATTACCACACCAGCATAGCTCACCGA-3′-NFQ-MGB) and *T. cati* (5′-HEX-TCTTTCGCAACGTGCATTCGGTGA-3′-NFQ-MGB)] and forward primers [*T. canis* (5′-GCGCCAATTTATGGAATGTGAT-3′) and *T. cati* (5′-ACGCGTACGTATGGAATGTGCT-3′)] and shared reverse primer 5’-GAGCAAACGACAGCSATTTCTT-3′) for both *Toxocara* species [12]. To assess the presence of PCR inhibitors in each sample, a commercially available internal amplification control (Internal DNA Extraction Control Kit; Primer Design, UK) was used according to the manufacturer’s instructions.

All of the reactions were performed in a total volume of 25 µL containing 1× SsoAdvanced Universal Probes Supermix (Bio-Rad), 5 µL template DNA, 0.5 µM of each primer and 0.25 µM of each labelled probe. Samples were tested in duplicate and run at 95 °C for 3 min, followed by 95 °C for 10 s and 60 °C for 30 s, for a total of 39 cycles. *Toxocara canis* and *T. cati* genomic DNA (45.0 and 7.13 ng/µL, respectively), extracted from adult worms, was used as positive controls. No template was used as a negative control. A sample was considered positive if there was detectable DNA at or before a cycle threshold of 38. If a negative internal control result was obtained for a particular well, the corresponding sample was reanalysed using 3 µL of DNA template instead of 5 µL, to minimise the presence of inhibitors.

### Assessment of field-grown spinach samples for* Toxocara* spp. eggs

Each 300-g spinach sample was processed as described above, with each pellet undergoing DNA extraction followed by multiplex qPCR analysis to investigate the presence of *Toxocara canis* or *T. cati* eggs. Each sample was tested in duplicate.

### Statistical analysis

Prevalence and its 95% confidence intervals (CIs) were calculated using EpiTools software [13]. Statistical analysis was undertaken using GraphPad software (https://graphpad.com/quickcalcs) and the chi-square test was used to compare *Toxocara* spp. prevalence between sites. A *P*-value < 0.05 was considered statistically significant.

## Results

### Recovery of eggs from spiked vegetable samples

In order to validate the sieving method, experiments were undertaken using 300-g samples of lettuce and spinach leaves spiked with known concentrations of *Toxocara canis* eggs. Positive qPCR results (Cq < 38) were obtained for all of the egg concentrations tested (4, 20 and 100 eggs/300 g). Positive control DNA samples yielded a Cq value < 15, negative control samples had a Cq of zero. The assay was able to effectively differentiate between *T. canis* and *T. cati* positive control DNA samples.

### Assessment of field-grown spinach samples for* Toxocara* spp. eggs

Spinach (*S. oleracea*) samples (120 in total), each comprising 300 g of leaves, were collected from four different commercial vegetable-growing farms in the south of England and processed using the sieving method followed by multiplex qPCR analysis.

In total, positive qPCR results were obtained for 30 samples, of which 28 were positive for *T. canis* and two were positive for *T. cati*. For two of the samples, positive *T. canis* results were obtained following repeat qPCR analysis using 3 µL of DNA template instead of 5 µL to minimise the effect of any inhibitors, as negative results were initially obtained for the internal amplification control. Repeat analysis yielded a positive result for both the sample DNA and the internal control DNA. Cq values obtained for the samples positive for *T. canis* ranged from 27.21 to 37.87, and for *T. cati* from 25.07 to 27.05. The overall *Toxocara* spp. prevalence was 25.0% (30/120) (95% CI 18.1–33.4%), and the species-specific prevalence was 23.0% for *T. canis* (28/120) (95% CI 16.7–31.7%) and 1.7% for *T. cati* (2/120) (95% CI 0.5–5.9%). The number of positive samples across the sampling sites and the associated prevalence values are shown in Table 1. There was a statistically significant difference in the number of positive samples obtained between farms (*χ*^2^ = 12.30, *df* = 3, *P* = 0.0064).

**Table 1:** The number of spinach (*Spinacia oleracea*) samples that tested positive for *Toxocara canis* or *Toxocara cati* eggs from each of the four farms sampled, and the resulting prevalence values

Farm identifier	No. of samples collected	No. of *T. canis-*positive samples	No. of *T. cati-*positive samples	Prevalence (%) of *Toxocara* sp. (95% CI)
1	33	15	0	45.5 (29.8–62.0)
2	25	6	0	24.0 (11.5–43.4)
3	35	5	2	20.0 (10.0–35.9)
4	27	2	0	7.4 (2.1–23.4)
Total	120	28	2	25.0 (18.1–33.4)

There was a statistically significant difference in the number of positive samples between farms (*P* = 0.0064)

*CI* Confidence interval

## Discussion

Whilst previous research has reported the presence of *Toxocara* spp. eggs on vegetables grown in small-scale community gardens [9], this is the first study, to our knowledge, in which vegetables grown on larger-scale, commercial farms in the UK have been tested for the presence of *Toxocara* spp. eggs. The finding of *Toxocara* spp. on spinach leaves grown on commercial farms is of concern, as this produce is readily available for purchase for consumption by a large number of people. Additionally, as spinach is not always cooked prior to consumption, the risk of consumers ingesting *Toxocara* spp. eggs is increased if the leaves are not washed thoroughly before they are eaten raw.

An overall prevalence of 25.0% was obtained across all four farms. However, there was considerable variation in prevalence between sites, with 45.5% the highest and 7.4% the lowest prevalence. This variation is reflected in the literature, with one study reporting prevalences of *T. cati* and *T. canis* on lettuce samples from Iran of 40.2% and 33.0%, respectively [10], and similarly in Libya, on 37% and 48% of lettuces sampled [14]. However, far lower prevalences have been reported in other studies. For example, in Switzerland, the prevalence of *Toxocara* spp. on lettuce was found to be 2.55% [11], and in Iran, values of 3.97% and 1.68% were found for ready-to-eat vegetables [15, 16]. In Turkey, the prevalence on lettuce and parsley samples was as low as 1.5% [17]. This variation in prevalence may be partially attributable to the different sampling approaches and testing methods used to recover eggs from vegetables between these studies. However, as demonstrated in the current study, there can also be wide variation in the degree of parasitic contamination of vegetables grown on farms in different locations.

In order for vegetable crops to become contaminated with *Toxocara* spp. eggs, definitive host species need to have access to farmland for the fields in which the crops are grown, or the water which is used to irrigate the crops, to become contaminated with eggs present in their faeces. Interestingly, the farm which had the highest *Toxocara* spp. prevalence in this study (farm 1) was the only one not to have a clear policy restricting members of the public from bringing their pet dogs onto the site. Whilst dog faeces can certainly contribute to environmental contamination with *Toxocara* spp. eggs, farmland is also commonly accessible to cats and foxes [18, 19]. Given that significantly more *T. canis* eggs were isolated in this study than *T. cati* eggs, and foxes are generally infected with *T. canis* rather than other *Toxocara* spp. [20], it is likely that foxes (and/or dogs) contribute more towards the environmental egg reservoir than cats at these sites. Perimeter fencing was absent from all the farms sampled, so the sites were easily accessible to a variety of animal species; there is also the possibility that the site owners had pets which had access to the farmland. With regards to the possible contamination of the water used to irrigate the crops, farms 1 and 3 used the mains water supply to water crops, farm 2 used water taken directly from a local river, and farm 4 utilised water from a self-built reservoir. Based on these limited observations, there did not appear to be an obvious association between water source and *Toxocara* spp. prevalence.

Another factor that may have impacted the contamination of spinach in this study was the weather conditions during sampling. The collection of samples at farm 1 took place following a period of heavy rain, which has been associated with an increased amount of soil splashing onto plants, particularly smaller crops with a maximum height of 50 cm [21]. The likelihood of any *Toxocara* spp. eggs present in the soil making their way onto the surface of the sampled leaves in this way was thus increased. For consistency, it is preferable to collect samples under the same climatic conditions to minimise variation in this regard. Since more intense rainfall events are predicted by climate warming scenarios, including in the UK [22], the risk of rain-splash dispersal of pathogens, including *Toxocara* spp., onto leafy vegetables, might increase in the future.

The presence of *Toxocara* spp. eggs was confirmed by means of qPCR testing. Whilst molecular approaches can increase the sensitivity of parasitic detection, one limitation of these methods is that they cannot show whether any of the eggs present are embryonated or viable, and thus infective. As microscopic determination of embryonation was not undertaken in the present study, it was impossible to know whether the eggs had the potential to cause infections in humans. If the eggs had been deposited by a definitive host within the 2-week period prior to sample collection, it is unlikely that they were embryonated [23]. Whilst embryonated eggs can persist in the environment for long periods of time under suitable conditions, and pose a risk of infection if consumed [24], it is difficult to know what conditions recovered eggs have been exposed to. It would be useful to determine whether recovered eggs have deformities, as these may indicate damage by desiccation and direct sunlight [25], which can have implications for their infectivity to humans.

Additionally, Cq values, obtained using molecular methods, only provide information on the quantity of DNA available in a sample, which can vary not only with the number of eggs present but also with their stage of development. The Cq value is not, therefore, directly correlated to the number of eggs in a sample. However, given that disease can occur in humans even with the migration of a single larva [26], the finding of *Toxocara* spp. DNA in food destined for public consumption is of concern. Further research incorporating microscopic analysis and enumeration of any eggs recovered would help to deepen our understanding of the risks of contaminated produce to public health.

The four farms sampled in this study were all located in the south of England. Although *Toxocara* spp. eggs were isolated from crops sampled at every site, care must be taken not to extrapolate these findings to the rest of the UK. Further research is needed to establish the prevalence of *Toxocara* spp. eggs on farms in other regions of the country.

Due to its availability on the farms sampled, only spinach was tested in this study. Being a leafy vegetable, it is more likely to be contaminated with parasitic eggs compared to non-leafy vegetables [10, 11]. Although washing has been shown to remove *Toxocara* spp. eggs from vegetables [27], studies on the effectiveness of different washing methods are lacking. Scientific evaluation of commercial washing methods for food safety has mainly been focused on bacterial pathogens [28, 29], and thus should be extended to helminths. It would also be useful to gather more data on other types of fruits and vegetables that grow close to the soil, to help in developing a clearer picture of the risk of food-borne transmission of *Toxocara* spp. in the UK.

## Conclusions

The results of this study demonstrate the presence of *Toxocara* spp. eggs on the leaves of spinach grown on commercial farms in the south of the UK for public consumption. *Toxocara* spp. was isolated from produce grown on every farm sampled, with a wide variation in the prevalence of *Toxocara* spp. eggs between farms. Given the potentially severe health risks associated with the ingestion of *Toxocara* spp. eggs, increasing public awareness of the importance of thoroughly washing vegetables before their consumption is crucial, particularly for vegetables that are often eaten without prior peeling or cooking. In addition, improving farm biosecurity to minimise access to farmland by definitive host species, and the thorough anthelmintic treatment of domestic definitive hosts that do have access, would decrease the risk of parasitic contamination of soil and also of crops at their point of origin.

## Data Availability

The data are presented in the published article.
